# Effect of Interaction Between Testosterone and Morphine on Serum Ghrelin Concentration in Sheep Fed on Different Dietary Energy Levels

**DOI:** 10.5812/ijem.4211

**Published:** 2012-06-30

**Authors:** Davood Jafaripour, Homayoun Khazali, Hasan Rokni, Hiva Alipanah

**Affiliations:** 1 Faculty of Biology Science, Shahid Beheshti University, Tehran, IR Iran; 2 Faculty of Physiology, Shahid Beheshti University, Tehran, IR Iran; 3 Applied Scientific Education Institute of Jahad Keshavarzi, Tehran, IR Iran; 4 Animal physiology, Shahid Beheshti University, Tehran, IR Iran

**Keywords:** Ghrelin, Morphine, Testosterone, Sheep

## Abstract

**Background:**

Ghrelin plays an important role in the regulation of food intake and body weight. It also decreases testosterone and opioid secretion.

**Objectives:**

The goal of the present study was to investigate the effect of testosterone, morphine or simultaneous injection of testosterone and morphine on mean serum ghrelin concentration in sheep.

**Materials and Methods:**

Ten sheep were divided into two groups (n = 5 in each group), they were fed with either 50 % or 100 % of their dietary energy needs for 10 days. Body weight was measured on the 1st and 10th day of the experiment. Animals in both groups received testosterone (60 μg/kg), morphine (0.15 mg/kg), or a simultaneous infusion of testosterone (60 μg/kg) and morphine (0.15 mg/kg), on the 8th, 9th, or 10th day of the experiment respectively. Blood samples were collected before and 2 hours after the infusions. Ghrelin concentration was determined by RIA (radio immunoassay).

**Results:**

In the 50 % group, ghrelin concentrations increased significantly on the 8th day of the experiment, compared to the 1st day (P < 0.05). While in the 100 % group, no significant change was observed. In both groups the animals’ body weight did not increase significantly on the 10th day compared to the 1st day. Testosterone significantly increased ghrelin levels after injection compared to before infusion, in both groups (P < 0.05). Morphine increased ghrelin concentration in both groups, but this increase was not statistically significant. Simultaneous injection of testosterone and morphine together, significantly increased ghrelin concentration following injection compared to before infusion, in both groups (P < 0.05).

**Conclusions:**

There is a direct correlation between food restriction, testosterone and ghrelin concentration in ruminants. However, a simultaneous injection of testosterone and morphine did not exert an additive effect on ghrelin secretion.

## 1. Background

Ghrelin, a 28 amino acid peptide with an n-octanoyl modification on Ser3, was first identified in the stomach as an endogenous ligand for growth hormone secretagogues receptors (GHSR-Ia) in 1999 (1). Ghrelin is well recognized as having an important role to play in the maintenance of energy homeostasis. It has been shown that one of the most important factors for the regulation of ghrelin secretion is nutritional status (2). So that, during fasting, ghrelin is secreted by cells in the stomach, neurons of the hypothalamus and in other tissues, and following food consumption mean plasma levels of ghrelin decrease (3). It has been established that ghrelin increases growth hormone secretion, food intake and body weight via GHSR-Ia (1). It also decreases energy expenditure and suppresses testosterone and endogenous opioid secretion (4, 5). There has not been any reports about the effects of testosterone or morphine on ghrelin secretion.

Also, it has been showed that testosterone, one of the most important hormones in balancing energy levels, increases food intake and body weight in rats (6). However, there are controversial results concerning the effect of opioids on food intake. Most of the studies report that opioid agonists exert a stimulatory effect on food intake. Therefore an injection of μ receptors agonists into the paraventricular (PVN) nucleus increases food intake and growth hormones, although a few of the studies have showed an inhibitory effect of opioid agonists on food intake (7-9).

## 2. Objectives

The goal of this study was to determine the effects of an intramuscular injection of testosterone or morphine on mean ghrelin concentration in sheep at different energy levels. Also, the effect of a combination treatment of testosterone and morphine was investigated on ghrelin concentration to determine whether a simultaneous infusion of these would have an additive effect on ghrelin secretion.

## 3. Materials and Methods

### 3.1. Animals

Zandi sheep (n = 10) weighing 35 ± 1Kg (provided by the research center of Khojir, Tehran, Iran) were housed under controlled temperature (25 ± 5°C) and light (12h light/dark cycle).

### 3.2. Injections and Blood Sampling

Ten sheep were divided randomly into two groups (n = 5 in each group). The first group received a diet at 100 % energy level and the second one received a diet at 50 % energy level for 10 days. Diets were formulated based on Agricultural and Food Research Council (AFRC) (10) (Table 1). During the course of the experiment, daily feed was weighed based on body weight and given to each animal individually every morning. The sheep had free access to fresh water. Diet 1 and 2 consisted of 100 % and 50 % of their maintenance energy requirements, respectively. Other requirements were balanced at maintenance level. Animals in both groups received an intramuscular injection of testosterone (6o μg/Kg), morphine (0.15 mg/Kg), or a simultaneous infusion of testosterone (6o μg/Kg) and morphine (0.15 mg/Kg), on the 8th, 9th, and 10th day of the experiment in a volume of 5ml at 0900-09:30 respectively. The doses of testosterone and morphine used in this study were chosen based on previous studies (11-14). Body weight was measured on the 1st and 10th day of the experiment. The blood samples (1ml) were collected daily via cannulae that were put into the jugular vein before and 2 hours after the infusions (15). Blood samples were centrifuged for 15 min at 3000 rpm and the serum stored at – 20°C until assayed for ghrelin concentration.

**Table 1:** Experimental Rations, Prepared Energy and Nutrients

Diet (Ingredients/Nutrition)	100% Energy	50% Energy
Wheat straw, g/day	10	260
Alfalfa (hay), g/day	50	50
Corn (grain), g/day	10	220
Corn gluten meal, g/day	210	85
Bone meal, g/day	1.34	0.47
Salt, g/day	1.66	1.22
Magnesium oxide, g/day	0.69	-
Vitamin and mineral supplement	3.50	3.50
Metabolizable energy, MJ/Kg	13.03	9.73
Crude protein, %	42.00	13.72
Calcium	0.52	0.24
Phosphorous, %	0.52	0.24
Sodium, %	0.45	0.21
Magnesium, %	0.24	0.11
Dry material intake, g/day	287	620
Metabolizable energy intake, MJ/Kg	3.74	6.03
Metabolizable protein intake, g/day	56.00	55.37

### 3.3. Hormone Assays and Statistical Analysis

The serum concentration of ghrelin was measured by ovine ghrelin kits (Mediagnost, Germany, provided by Tabeshyarnor Company, Iran) and the method of a homologous double antibody - immunoassay (RIA). Sensitivity, intra-assay and inter-assay of the ghrelin kits were 0.04ng/ml, 5 % and 7.6 % respectively. The results are presented as mean ± SEM. The data were analyzed by paired T- test, unpaired T- test and SPSS Software (version 16). In all cases P < 0.05 was considered to be statistically significant.

## 4. Results

**Figure 1:**
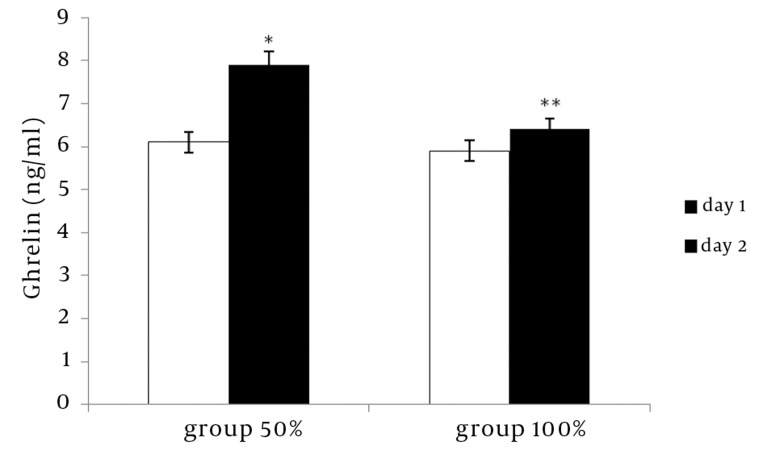
The Effect of Food Restriction on Mean Ghrelin Concentration in Groups with 50 % or 100 % Energy Level (P < 0.05). * comparison between day 1 and 8; ** comparison between 50 % and 100 % groups on the 8th day.

The results showed that the mean ghrelin concentration of the serum increased significantly in the group at the 50 % energy level (P < 0.05) on the 8th day of experiment compared to the first day. In the group with a 100 % energy level diet, no significant changes were observed in ghrelin concentration levels (Figure 1). Also, on the 8th day of the experiment, a significant difference was observed in the mean ghrelin concentration levels between the 50 % and 100 % energy diet conditions (Figure 1) (P < 0.05). In both groups, body weight had not increased significantly on the 10^th^ day of experiment compared to the first day (Table 2).

**Table 2:** Mean Body Weight in Group 100 % or 50 % in First and 10th Day of Experiment.

Group	1^st^ Day, Kg	10^th^ Day, Kg
100 %	35.68	36.75
50 %	35.37	34.81

**Figure 2:**
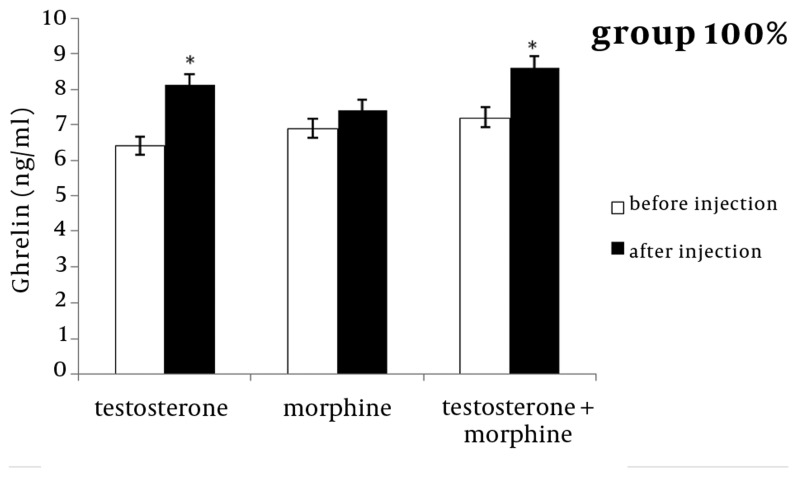
The Effect of Testosterone, Morphine or Simultaneous Infusion of Testosterone and Morphine on Mean Ghrelin Concentration Before and After Injections in Group with 100 % Energy Level (P < 0.05)

**Figure 3:**
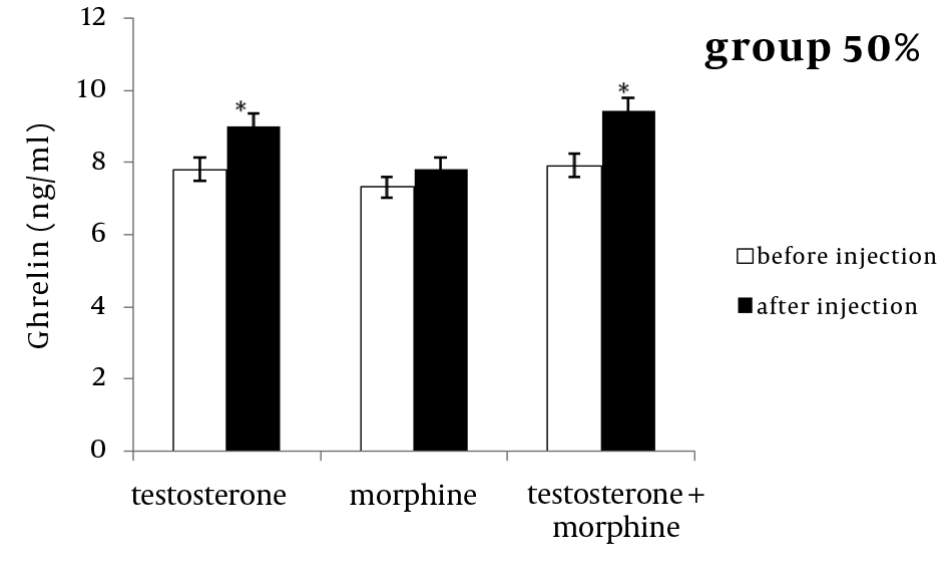
The Effect of Testosterone, Morphine or Simultaneous Infusion of Testosterone and Morphine on Mean Ghrelin Concentration Before and After Injections in Group with 50 % Energy Level (P < 0.05).

In the 50 % or 100 % groups, testosterone significantly increased ghrelin concentration after the infusion compared with levels before the injection (P < 0.05) (Figure 2 and Figure 3). In both groups, morphine increased ghrelin concentration after infusion compared to before injection, but this increase was not statistically significant in either group (Figure 2 and Figure 3).

The results of the simultaneous infusion of testosterone and morphine also revealed that, ghrelin concentrations increased significantly in both groups after infusion, compared to before the sheep were injected (P < 0.05) (Figure 2 and Figure 3). Simultaneous injections of testosterone and morphine did not exert an additive effect on ghrelin secretions compared to the testosterone group.

## 5. Discussion

In this study, we demonstrated that food restrictions significantly increased mean ghrelin concentration in sheep. The results of this study were consistent with previous studies which reported that ghrelin concentration increased during fasting or food restriction and decreased with food consumption in both rodents and mammals (2, 3). However, the regulatory factors of ghrelin secretion are not completely clear, but it is thought that blood glucose or fatty acid levels could be one of the most important factors. It has been shown that oral consumption or intravenous injection of glucose or fatty acids decreases ghrelin secretion (16-18). So, food restriction may increase ghrelin secretion partly via decreasing blood glucose or fatty acid levels. Also, we demonstrated that testosterone significantly increased ghrelin concentrations in sheep that were fed at a 50 % or 100 % energy level. In the present study the effect of testosterone on ghrelin secretion was investigated for the first time in ruminants, but the precise mechanism for its action on ghrelin secretion needs further studies, as it has been shown that testosterone increases food intake partly by increasing NPY (19) and ghrelin exerts its stimulatory effects on food intake by increasing NPY and AgRP (20). It has also been revealed that there is a reverse correlation between testosterone and leptin concentrations (21). Therefore, one could infer that testosterone may partly increase NPY concentration and food intake via an increase in ghrelin levels and a decrease in leptin secretion.

There is a complex relationship between opioids and the neurotransmitters systems which regulate food intake. It has been shown that the interaction between NPY, ghrelin or AgRP (agouti related peptide) and opioids plays an important role in the regulation of food intake (21, 22). NPY or AgRP neurons in the arcuate nucleus (ARC) of the hypothalamus increase food intake in all species, including sheep. It has been established that the effect of NPY or AgRP on appetite is mediated via opioid receptors (21, 22). In the present study, the effect of morphine on ghrelin concentrations was investigated in ruminants. Based on previous studies, subcutaneous, intraperitoneal or intramuscular injections of morphine, or other opioid receptor agonists, can influence energy balance and food intake, both in rodents and in humans. In fact, the effect of opioids on food intake remains controversial. Some studies have reported that low doses of opioid receptor agonists significantly increased food intake, while high doses decreased food intake in rats. Also, some studies reported a neutral effect of different opioids on food intake (8, 9, 13, 14, 23). However, the dose of morphine used in this study did not significantly alter ghrelin secretion levels, so in order to determine the precise effects of opioids, different doses of morphine should be used. The molecular mechanism of morphine effects on food intake should also be investigated to determine whether opioids exert a direct effect on the expression of the neuropeptides involved in food intake regulation, or if it acts as a moderator in mediating the effect of other peptides or hormones on food intake.

Some hormones or drugs when used alone may exert only a small effect on this special pathway, however, when they are used in conjunction with other drugs or hormones they may have a much greater effect. On the other hand they may exert a synergistic effect. In this study we showed that an injection of morphine by itself did not have any significant effect on ghrelin concentration, while an injection of testosterone significantly increased ghrelin secretion. So, we need to examine the synergistic effect of testosterone and morphine on mean ghrelin concentration. The results showed that a simultaneous infusion of testosterone and morphine together significantly increased ghrelin concentration, but a simultaneous infusion does not have an additive effect on ghrelin levels. The present study investigated the effect of a combination treatment of testosterone and morphine on ghrelin secretion for the first time, but further studies are still needed to determine the possibility of the effect of an interaction between them on ghrelin secretion in ruminants, because morphine may act as a mediator for the regulation of energy balance as it has been shown that the effect of NPY or AgRP on the appetite was mediated via the opioid receptors (21, 22). Consequently, testosterone may exert its stimulatory effect on ghrelin secretion and thereby food intake through the mediatory effect of morphine.

In summary, the results of this study revealed that food restriction and testosterone significantly increased mean serum ghrelin concentration in ruminants. However, a simultaneous injection of testosterone and morphine did not exert an additive effect on ghrelin secretion. Morphine could prove to have a role in mediating the effect of testosterone on ghrelin secretion and this warrants further investigation.
